# Effectiveness of a progressive resistance exercise program for industrial workers during breaks on perceived fatigue control: a cluster randomized controlled trial

**DOI:** 10.1186/s12889-020-08994-x

**Published:** 2020-06-03

**Authors:** Hélio Gustavo Santos, Luciana Dias Chiavegato, Daniela Pereira Valentim, Rosimeire Simprini Padula

**Affiliations:** 1 Master and Doctoral Program in Physical Therapy, Universidade Cidade de São Paulo (UNICID), Rua Cesário Galeno, 448/475 Tatuapé, São Paulo, 03071-000 Brazil; 2 São Camilo University Center, Cachoeiro de Itapemirim, Espírito Santo, Brazil; 3 Pulmonology Division, Universidade Federal de São Paulo, São Paulo, Brazil; 4 Department of Physical Therapy, Universidade Cidade de São Paulo, São Paulo, Brazil

**Keywords:** Occupational health, Employees, Physical activity, Health promotion

## Abstract

**Background:**

There is strong evidence that physical exercise in the workplace is effective for reducing workers’ musculoskeletal complaints. Studies with industrial workers and studies on progressive resistance exercises during breaks are scarce. Our aim was to evaluate the effects of a resistance exercise program on perceived fatigue control among industrial workers.

**Methods:**

204 employees from the dairy industry were allocated to two groups, the intervention group (IG) (*n* = 98) and the control group (CG) (*n* = 106). The primary outcome measures were perceived fatigue control and maximum muscle strength, measured through the Need for Recovery Scale and one-repetition maximum contraction (1-RM), respectively. Secondary outcome measures were musculoskeletal complaints, physical activity level, perceived risk factors, physical fitness (BMI, vital signs, and body fat percentage), and workers´ productivity. All outcomes were assessed at baseline and then again after 4 months. The IG performed resistance exercises using progressively greater loads while the CG performed general exercise using elastic bands. The exercise protocols were performed three times per week for 20 min. An intention-to-treat analysis was performed using the mixed linear model. Results were considered significant when *p* < 0.05.

**Results:**

The IG did not show to be superior to the CG, although both groups improved perceived fatigue control and muscle strength after the resistance physical exercise program in the worplace. There was also no significant difference between the groups for musculoskeletal complaints and other secondary variables analyzed. However, both groups showed significant improvements between baseline and after 4 months of intervention for all evaluated outcomes (*p* < 0.05).

**Conclusion:**

The implementation of a progressive resistance exercise program during work breaks for perceived fatigue control was no more effective than exercises using elastic bands. However, resistance exercises during work breaks presented better results on all measured outcomes regardless of the exercise protocol used.

**Trial registration:**

U.S. National Institutes of Health, ClinicalTrials.gov Identifier: NCT02172053. Registered 19 June 2014.

## Background

Fatigue is a common symptom among workers due to high work demands, whether they are physical demands, affecting the musculoskeletal system, or mental health demands that lead to errors and accidents at work, both of which consequently reduce the individual’s ability to work [1–3]. In general, it is normal for the individual to need to rest after a day’s work to reverse the acute fatigue. However, if over the course of days this rest is not sufficient for recovery of the individual, there is an accumulation of fatigue that leads to overload, which can lead to more serious disease conditions and even chronic fatigue [3–5]. The effects of fatigue on workers in the short and long term are musculoskeletal injuries and non-communicable diseases in general, as well as anxiety and depression [3, 4]. The contributory factors for the worsening of this condition are inadequate life habits, long working hours, insufficient sleep, and lack of physical exercise [6].

Studies has demonstrated several interventions implemented in the workplace to promote the health and safety of workers [7–9], but the most effective are exercise-based [9]. While most of these studies have been conducted with office workers who have different job demands than production workers, there is strong evidence for the positive effect of physical exercise in the workplace for all workers group [9–14]. A variety of training protocols have been tested in the workplace, including light training without resistance, stretching, relaxation exercises, light aerobic and dynamic exercises, and resistance training using dumbbells, isokinetic equipment, elastic bands, and exercises against gravity [12–15]. Resistance training has been shown to be the most effective for reducing musculoskeletal complaints and perceived physical effort [12–18].

The training protocols described in the literature [13–15] using strength, resistance, or light resistance training, all of which have shown benefits of maintaining strength [19]. Resistance exercises are more efficient than light training since they improve the locomotor apparatus functions and have positive impacts on the cardiovascular and musculoskeletal systems [13, 16].

Recommendations based on systematic reviews indicate that resistance exercises in the workplace are effective at reducing musculoskeletal complaints when performed in 20-min sessions at least three times a week for 10 weeks or more [13, 17, 18]. However, few studies of physical exercise during the workday have been designed for controlling perceived fatigue [12] in industrial workers [13, 16]. Only one study compared the effect of a progressive resistance exercise program having better results than a usual physical exercise program on forearm pain and work disability in industrial workers [16]. Currently, no other studies compare the effects of resistance training protocols in the workplace on issues such as perceived fatigue, musculoskeletal complaints, and general health.

Therefore, this study aimed to compare two resistance exercise protocols performed in an industrial environment during the workday and assess which of these programs is most effective for controlling perceived fatigue and increasing muscle strength among workers. The intervention group in our study performed progressive resistance physical exercise, while the control group completed a minimal intervention exercise with constant load using an elastic band.

In order to reduce the possibility of exhausting the participants, the exercise protocols were intended to be less intense than typical resistance training programs, a feature that we have also observed in other studies, since combining the fatigue associated with a regular work day and the fatigue associated with an intense workout may make it more difficult to observe the potential positive effects of the intervention [19].

Our hypothesis was that the intervention group will have better perceived fatigue control and greater gains in muscle strength than the control group, associated with the use of progressively greater loads. However, as has been shown in previous studies, both groups could experience an improvement in perceived fatigue control and muscle strength due to the resistance exercises.

## Methods

### Study design and ethical approval

This is a cluster randomized controlled trial (RCT) with double-blinding, parallel group design, and prospective registration. The ethics committee at Universidade Cidade de São Paulo (Process Number – 454709) approved the study. This study has been reported according to the CONSORT Statement guidelines.

### Setting, recruitment, and eligibility criteria for participants

For this study, 352 workers from thirteen sectors of a medium-sized dairy plant were invited. The beginning of the study involved the approval of the project by the company’s managers. In the next step, the researchers printed the materials to publicize the exercise program to all workers in the production area. An inaugural lecture was held for 2 days (20 min for each group of 80 workers). The workers were informed about the importance of physical exercises in the workplace. Emphasis also was placed on changes in lifestyle (physical exercise, sleep quality, healthy eating). The lectures were held in three work shifts for all workers.

The participants were included according to the following eligibility criteria: workers allocated in production sectors, both sexes, aged 18–65 years, not outsourced or temporary, and no restrictions from the medical department. The participants of the study signed an informed consent form. The study occurred from June 2015 until December 2016 in Espirito Santo, Brazil. More information about eligibility criteria and methods as well as exercise protocol details were published previously [20].

### Randomization and blinding

The participants were cluster randomized by production sector (*n* = 13). Particpants enrolled by sector: Boilers (*n* = 23), Receiving/Cooling/Standardization (*n* = 26), Manufacturing (*n* = 24), Butter (*n* = 24), Cheese (*n* = 33), Milk Caramel (*n* = 24), Yoghurt (*n* = 22), Creamy Cheese (*n* = 25), Ultra-high Temperature Plant (*n* = 60), Milk Powder (*n* = 25), Stock (*n* = 24), and Warehouse (*n* = 22). The randomizations were performed after occupational musculoskeletal exposure classification by Quick Exposure Check (QEC) and accounting for the general demands of each task [21, 22]. The production sectors with moderate and low occuopational demands were equally allocated to intervention and control group. The majority of the sectors were classifyied by QEC as having moderate musculoskeletal exposure for biomechanical risks. The Yoghurt, Butter, Boilers, and Warehouse sectors presented low biomechanical risk factors.

Cluster randomization was necessary to ensure that sectors with different levels of exposure were distributed in both groups. The program “Research Randomizer” (http://www.randomizer.org) was used to randomize the clusters. The randomization was performed by a researcher who was not involved in any other stages of the study. The double blinding was possible because both groups performed physical exercise three times a week on alternate days with training consisting of three sets of ten repetitions with a 30-s interval between sets; the only difference between groups were the training requirements. The participants were blind to type of exercise each group received because both performed exercise in the workplace. Likewise, the data collectors were unaware of the intervention the participants received.

### Sample size calculation

To determine the sample size, the difference between need to recovery of 123 workers at the beginning and end of the workday during 7 days was evaluated. The sample size was calculated to detect a difference of 20% in measured outcomes between groups, α = 0.05 and statistical power of 80% required a minimum of 86 workers per group.

### Procedure

First, all the workers attended a lecture regarding the importance and benefits of practicing physical exercise in an industrial workplace. Emphasis was placed on changes in lifestyle (physical exercise, importance of sleep, and adequate nutrition) within and outside work, lasting 20 min. They also received an explanation of the objectives of the study. After this step, those who agreed to participate in the study answered the questionnaires and underwent a physical fitness evaluation to identify their general health conditions, perceived fatigue control, and muscular streght [20]. The outcome measures for all workers were performed at baseline and at a follow-up 4 months later. The intervention and control groups performed the physical exercise programs for 4 months (18 weeks), three times per week. Ten trained physiotherapists and physical educators not affiliated with the company supervised and guided the physical exercise sessions at all times. All occurrences during the physical exercise program period and adherence of participants were recorded in a notepad [20]. The adherence was calculated according to the participant’s attendance of the sessions in relation to the total number of sessions (54 sessions). The physical exercise groups included 8–10 participants at a time in the workplace.

### Interventions

All participants received education about health self-management, the benefits of physical exercise and a healthy lifestyle, as well as guidance on the adjustment of workstations. The instructors involved in the study gave the lectures on 2 days (topics included the impact of fatigue, work demands, and rest breaks, the setup of workstations, and the importance of exercise in the workplace).

### Progressive resistance exercise (PRE) – intervention group

The intervention group performed progressive resistance exercises starting with 30% of the one-repetition maximum (1 RM - kg) value, an appropriate amount of resistance for an exercise program in the workplace. The materials used for resistance training were dumbbells and barbells. The progression of the load increase occurred through the analysis of the individual’s adaptation [19]. The muscle groups trained were elbow flexors, elbow extensors, trunk flexors, trunk extensors, knee flexors, knee extensors, thigh adductors, thigh abductors, and ankle dorsal and plantar flexors.

### Compensatory workplace exercise (CWE) – control group

The participants of the control group performed compensatory exercise that is a usual physical exercise program in the workplace in Brazil. The compensatory exercise is frequently done during breaks to compensate for the effect of physical demands on the musculoskeletal system. The protocol used consisted of an exercise program using stretching, movements with gravity as resistance, or movements with elastic bands with medium resistance (medium resistance being considered 1.7–2.6 kg with dimensions 7.5 m × 0.6 cm, adjusted for the individual) for all major muscle groups [20].

For both the intervention and control groups, the exercise was carried out three times per week, 20 min per session [13]. Each exercise was performed in three sets of ten repetitions with 30 sec intervals between sets [20].

The resistance training for the muscle groups of the upper limbs, trunk, and lower limbs was conducted 3 days per week, with an average of four exercises per day.

At the beginning of each session and after the physical exercise program, the participants’ heart rate and blood pressure parameters were measured and monitored. If important changes in vital signs were noted before or in response to physical exercise, participants were referred to the medical department [23].

### Outcomes measure

#### Primary outcomes

Fatigue outcomes were assessed through the perception of fatigue and muscular fatigue.

1. Perceived fatigue control by workers was measured using the Need for Recovery Scale (Br-NFR) that demonstrated to be reproducible and valid in cross-cultural adaptation to Brazilian-Portuguese [24]. The Br-NFR scale has been used to assess the need for recovery from work-induced both mental and physical fatigue. The scale has eleven items such as: ‘*At the end of a working day I am really feeling worn out’* and *‘I have trouble concentrating in the hours off after my working day’*. A total score ranging from zero (lowest) to 100 (maximum). A higher score indicates greater need for recovery due to work demands [24, 25].

2) Maximum Muscle Strength was tested by one-repetition maximum (1-RM - kg) in the biceps, triceps, deltoid, quadricepsfemoral, hamstrings, and triceps sural muscle groups using an appropriate load for each participant [19].

#### Secondary outcomes

1. At baseline, the Nordic Musculoskeletal Questionnaire (NMQ) has good reliability and is valid to evaluate musculoskeletal complaints during the last 12 months and last 7 days [26]. The Pain Numeric Rating [27], with a likert scale (zero means “no pain” and ten means “the worst possible pain”) was used to evaluate pain intensity by body region in the last 24 h.
2. The Baecke Physical Activity Questionnaire is reliable and valid to measure habitual physical activity in Brazilian adult. It was used to evaluate level of physical activity of participants. The questionnaire determines the occupational physical activity level (PAO), sport and exercise in leisure time (ESL), and exercise in leisure and locomotion (ELL). The questionnaire score ranges from zero to ten points [28].

3) The Job Factor Questionnaire was tested on Brazilian proving to be reproducible and valid to evaluate the risk factors perceived by workers that could be associated with the development of musculoskeletal complaints [29]. Each risk factor (fifteen are listed - *1) Performing the same task over and over; 2) Working very fast for short periods (lifting, grasping, pulling,* etc.*)* ranges from zero points (indicating “no problem”) to ten points (indicating the “largest possible problem”) [29].

4) A physical fitness assessment was performed to evaluate a) Body Mass Index (BMI) from measures of weight and height; b) Vital signs including heart rate, blood pressure, respiratory rate, and oxygen saturation; and c) Body fat percentage. Vital signs were measured using a heart monitor (POLAR - RS800CX), a fingertip pulse oximeter, a stethoscope, and a sphygmomanometer. Body fat percentage was assessed using body skinfolds from the biceps, triceps, pectoralis, subscapularis, midaxillary line, suprailiac skinfold site, abdomen, thigh, and calf.
5) The Health and Work Performance Questionnaire (HPQ) was used to evaluate the workers’ productivity. The HPQ Brazilian Portuguese version proved be valid and reliable [30]. The scale asks respondents to rate their productivity with the using scores range from zero (worst performance) to ten (top performance) points, *‘How would you rate your usual job performance over the past during the last three months?’* [30].

### Statistical analysis

The statistical program SPSS Statistics version 24.0 software (IBM, USA) was used for all data analyses. To test the normality of data, we used the Shapiro-Wilk test. Descriptive analysis (frequencies, means, standard deviation, differences, and confidence interval) was performed for all collected variables. The intention-to-treat principle was used to analyze the results. The difference between the groups (intervention and control) was analyzed using a linear mixed model test. The McNemar’s test was used to compare differences between groups on categorical variables. Results were considered significant when *p* < 0.05.

## Results

**Fig. 1:**
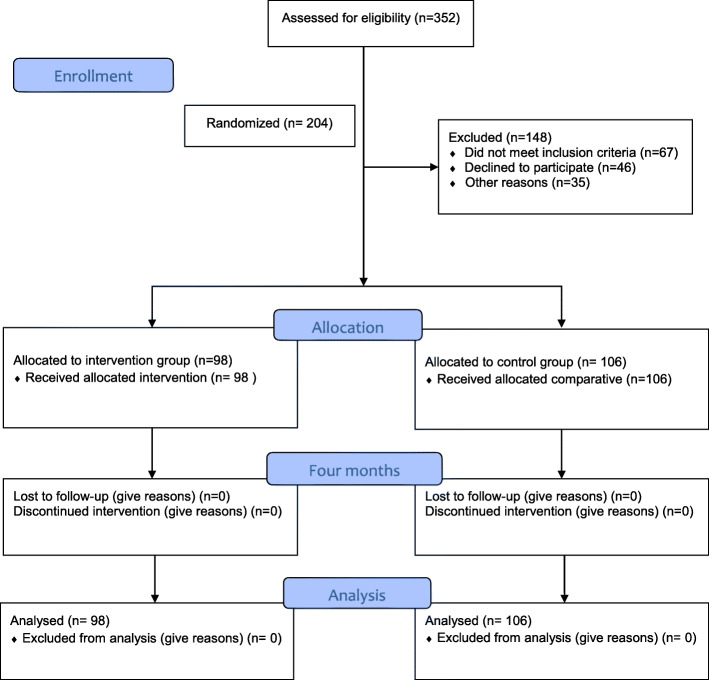
Study flow diagram of participants. ^a^All workers lost to follow-up were dismissed due to business reasons

204 eligible workers were recruited from thirteen production sectors. The clusters were randomly assigned to the intervention (*n* = 98, 6 clusters) and control (*n* = 106, 7 clusters) groups. Figure 1 shows the flowchart of recruitment, participants, allocation to groups, and follow-up.

### Participant’s characteristics

The participants characteristics at the baseline were predominantly male (83.3%) with an average time on the job of 53.95 (13.9) months. The mean age of the workers was 36.02 (12.4) years with a predominance of married individuals (54%). The somatotypes of the workers were, for both groups, mostly classified as mesomorphs (high percentage of lean mass) and endomorphs (high percentage of fat). The sociodemographic characteristics of the participants are presented in Table 1.

**Table 1:** Participants’ characteristics at baseline (*n* = 204)

	Groups	
Variables	Intervention *n* = 98	Control *n* = 106
**Age (years), mean (SD)**	34.3 (11.9)	37.74 (12.9)
**Gender, n (%)**		
Female	20 (20.4)	14 (13.2)
Male	78 (79.6)	92 (86.8)
**Marital status, n (%)**		
Married	53 (54.1)	55 (51.9)
Single	38 (38.8)	44 (41.5)
Divorced	6 (6.1)	5 (4.7)
Widowed	1 (1)	2 (1.9)
Education level*, n (%)		
Medium and high	50 (51)	59 (55.7)
Low	48 (49)	47 (44.4)
**Time on the job (months), mean (SD)**	53.2 (14.2)	54.7 (13.6)
**Somatotype, n (%)**		
Endomorph	32 (32.7)	38 (35.8)
Mesomorph	54 (55.1)	46 (43.4)
Ectomorph	12 (12.2)	22 (20.8)

*SD* Standard Deviation. * Medium and high education (high school and university); Low education (elementary degree)

### Primary outcomes measure

Training loads for the intervention group were increased by 20% during the 4 month period.

Table 2 presents the results of the baseline and final evaluations of perceived fatigue control and muscle strength (1 RM). The results showed that the intervention group did not show better results than the control group for the fatigue outcomes (perceived fadigue control and muscle strength). Although both protocols brought benefits to participants and so both interventions were effective (Table 2).

**Table 2:** Results at baseline and four months for intervention and control groups (*n* = 204)

Primary Outcome Measure		Groups Unadjusted, Mean (SD)		Unadjusted Within-Group, Mean Difference (95% CI) [Baseline minus four months]		Between-Group Adjusted, Mean Difference (95% CI)
		Intervention (*n* = 98)	Control (*n* = 106)	Intervention (*n* = 98)	Control (*n* = 106)	
**Need for Recovery** **0–100**	Baseline	68.4 (12.1)	56.2 (10.9)			
	Four months	59.4 (8.7)	51.7 (8.6)	9.93 ^a^ (7.3 to 12.5)	6.77 ^a^ (5.78 to 7.75)	7.7 (−6.2 to 12.4)
**Muscle Strength** **1-RM (kg)**						
Biceps	Baseline	16.6 (2.3)	16.5 (2.5)			
	Four months	17.8 (2.5)	17.8 (2.5)	- 1.2 ^a^ (− 1.4 to − 1.0)	- 1.3 ^a^ (− 1.4 to − 1.0)	0.0 (0.0)
Triceps	Baseline	11.1 (1.9)	11.2 (2)			
	Four months	12.4 (2.2)	12.3 (2)	- 1.3 ^a^ (−1.4 to − 1.0)	- 1.1 ^a^ (− 1.4 to − 1.0)	0.0 (− 0.5 to 0.5)
Deltoid	Baseline	8.8 (1.6)	8.9 (1.4)			
	Four months	10 (1.9)	10.1 (2)	- 1.2 ^a^ (−1.3 to − 1.0)	- 1.2 ^a^ (− 1.3 to − 1.0)	0.1 (− 0.5 to 0.3)
Quadriceps femoris and Hamstrings	Baseline	27.4 (2.4)	27.7 (2.3)			
	Four months	29.7 (3.4)	30.3 (3.3)	- 2.4 ^a^ (− 2.7 to − 2.0)	- 2.6 ^a^ (− 2.9 to − 2.3)	0.4 (− 1.1 to 0.2)
Triceps surae	Baseline	27 (2.4)	27.4 (2.5)			
	Four months	29.1 (3.0)	29..3 (3.0)	- 2.1 ^a^ (−2.2 to −1.7)	- 1.9 ^a^ (− 2.2 to − 1.7)	0.2 (− 1.0 to 0.3)

^a^Significant difference between groups (*p* < 0.05); SD - standard deviation; 1 RM - one repetition maximum. There were no missing data

### Secondary outcomes measure

Table 3 shows the prevalence of workers with musculoskeletal symptoms at baseline and after 4 months. There was a significant reduction of complaints per body segment for both groups (*p* < 0.05).

**Table 3:** Results at baseline and four months regarding musculoskeletal symptoms for intervention and control groups (*n* = 204)

Musculoskeletal symptoms, n (%)	Baseline		Four months		
	Intervention (*n* = 98)	Control (*n* = 106)	Intervention (*n* = 98)	Control (*n* = 106)	*p*-Value
Neck	32 (32.7)	37 (37.8)	10 (10.2)	15 (14.2)	0.03 *
Shoulders	30 (30.6)	33 (33.7)	5 (5.1)	7 (6.6)	0.00 *
Upper back	27 (27.6)	33 (33.7)	6 (6.1)	10 (9.4)	0.01 *
Elbows	12 (12.2)	10 (10.2)	8 (8.2)	11 (10.4)	0.06
Wrists/hands	22 (22.4)	25 (25.5)	1 (1.0)	3 (2.8)	0.00 *
Low back	44 (44.9)	31 (31.6)	6 (6.1)	7 (6.6)	0.00 *
Hips/thighs	6 (6.1)	5 (5.1)	0 (0.0)	0 (0.0)	0.36
Knees	30 (30.6)	32 (32.7)	5 (5.1)	11 (10.4)	0.00 *
Ankles/feet	13 (13.3)	22 (22.4)	2 (2.0)	2 (1.9)	0.11

*Significant difference within-groups (*p* < 0.05). There were no missing data

Table 4 shows the effects of the interventions for the outcomes: level of physical activity, perceived risk, physical fitness (BMI, blood pressure, heart rate, and oxygen saturation), and productivity. The results showed that the intervention group had no more improvement of these outcomes the than control group. However, there was an improvement of these outcomes after 4 months for both groups.

**Table 4:** Results of intervention on the secondary outcomes: physical activity level, perceived risk factor, physical fitness, and productivity at baseline and four months (*n* = 204)

Secondary Outcomes		Groups’ Unadjusted Means		Unadjusted Within-Group Differences, 95% CI [Baseline minus four months]		Between-Group Adjusted Mean Differences, 95% CI
		Intervention (*n* = 98)	Control (*n* = 106)	Intervention (*n* = 98)	Control (*n* = 106)	
Pain intensity (0–10 points)	Baseline	3.0 (3.7)	1.8 (3.0)			
	Four months	0.4 (1.4)	1.3 (2.6)	2.6^a^ (1.9 to 3.3)	0.5^a^ (0.1 to 0.9)	2.13^b^ (1.4 to 3.0)
Physical activity level^c^ (0–10 points)	Baseline	8.4 (0.8)	8.4 (0.7)			
	Four months	9.5 (0.6)	9.5 (0.7)	−1.1 ^a^ (−1.2 to −1.0)	−1.1 ^a^ (−1.2 to −1.0)	0 (−0.1 to 0.1)
Perceived risk-factors for musculoskeletal pain (0–150 points)	Baseline	58.2 (33.2)	52.6 (35.2)			
	Four months	48.7 (25.7)	44.4 (28.5)	9.5 ^a^ (8.2 to 10.8)	8.2 ^a^ (6.93 to 9.47)	4.9 (−3.5 to 13.4)
Physical Fitness						
BMI (kg/m^2^)	Baseline	25 (4.2)	25.3 (3.9)			
	Four months	24.6 (4.0)	24.8 (3.7)	0.4 ^a^ (0.3 to 0.5)	0.5 ^a^ (0.4 to 0.6)	0.2 (−1.3 to 0.8)
Blood pressure systolic (mmHg)	Baseline	121.2 (15.5)	122.2 (13.9)			
	Four months	116.9 (10.8)	118.5 (8.5)	1.2 ^a^ (−1.8 to 4.4)	1.2 ^a^ (− 1.8 to 4.4)	0 (−0.1 to 0.1)
Blood pressure diastolic (mmHg)	Baseline	76.8 (9.3)	76.6 (10.8)			
	Four months	75.4 (7.8)	75 (9.8)	1.5 ^a^ (−0.8 to 2.2)	1.5 ^a^ (− 0.8 to 2.2)	0.3 (−2.2 to 2.8)
Heart rate (bpm)	Baseline	75.2 (11.6)	77.5 (11)			
	Four months	69.5 (7.3)	70.7 (6.7)	6.2 ^a^ (5.2 to 7.2)	7.2 ^a^ (6.2 to 8.2)	1.7 (0.7 to 4.1)
Respiratory rate (rpm)	Baseline	19.7 (3.7)	20.1 (3.7)			
	Four months	19 (2.2)	19.4 (2.2)	0.7 ^a^ (0.3 to 1.0)	0.5 ^a^ (−0.1 to 0.9)	0.4 (−1.1 to 0.3)
Oxygen saturation (%)	Baseline	97.8 (1.2)	97.8 (1.1)			
	Four months	98.2 (0.7)	98 (0.8)	−0.3 ^a^ (− 0.4 to − 0.2)	− 0.2 ^a^ (− 0.3 to − 0.4)	0 (− 0.3 to 0.17)
Productivity (0–10)	Baseline	7.2 (0.8)	7.2 (0.8)			
	Four months	6.1 (0.7)	6.0 (0.7)	1.2 ^a^ (1.2 to 0.9)	1.2 ^a^ (1.2 to 0.9)	0 (− 0.1 to 0.1)

^a^Significant difference within groups (*p* < 0.05). ^b^Significant difference between groups (*p* < 0.05). ^c^Physical Activity Level = PAO + ESL + ELL (physical activity at occupation (PAO), sport and exercise in leisure time (ESL), and exercise in leisure and locomotion (ELL)). *SD* standard deviation, *BMI* body mass index, *bpm* beats per minute, *rpm* respirations per minute. There were no missing data

The body fat percentage reduced significantly after 4 months for both groups (Table 5).

**Table 5:** Results of intervention on the secondary outcome: skinfolds (body fat percentage) for intervention and control groups (*n* = 204)

Body fat percentage n (%)	Baseline		Four months		
	Intervention (*n* = 98)	Control (*n* = 106)	Intervention (*n* = 98)	Control (*n* = 106)	*p*-Value
Triceps	14.1 (6.4)	12.7 (5)	12.8 (5.2)	11.9 (4.1)	0.02
Biceps	10.2 (4.9)	9 (4.6)	9.5 (4.1)	8.4 (3.5)	0.00
Pectoral	12.1 (5)	11.6 (4.9)	11 (4.3)	10.8 (4.4)	0.02
Midaxillary line	14 (5.9)	13.2 (5.5)	12.9 (5.2)	12.1 (4.5)	0.01
Subscapular site	17.8 (8.4)	18.4 (7)	16.3 (7)	17.2 (6)	0.03
Abdominal	21.5 (9.6)	22.1 (7.4)	19.9 (8.3)	21.1 (6.8)	0.03
Suprailiac site	15.4 (6.9)	15.3 (6.2)	14.5 (6.5)	14.3 (5.8)	0.01
Thigh	17.7 (10.1)	17.3 (7.8)	16.7 (8.9)	16.6 (7.1)	0.01
Calf	13.3 (7.3)	13 (6.8)	13 (7)	12.4 (5.9)	0.00

Significant difference within-groups (*p* < 0.05). There were no missing data

The present study obtained a high adherence of participants to the physical exercise programs (*n* = 204; 100%). In fact, 77.6% of the workers participated in the exercise training three times per week. Of the remaining 22.4%, only one to two absences during 1 or 2 weeks of the intervention were reported.

## Discussion

The workplace is recognized as an ideal setting for the implementation of health promotion through physical exercise, with a minimum of 1 h per week, mainly because it involves individuals who do not have the time or have other obstacles to participating in physical exercise outside the workplace [31]. In addition, the World Health Organization [32] has also emphasized that the workplace is an especially good place for health promotion programs, besides being an ethical and social commitment of the companies. In addition, health promotion actions that include physical exercise are potentially effective in improving workers’ lifestyles [33].

Despite strong evidence that physical exercise has very positive effect for workers, it is still very difficult to implement and maintain health promotion programs in the workplace. It is even more difficult to assess the effectiveness of these programs through scientific studies. Faced with the challenges of conducting a controlled clinical trial in occupational health in an industrial environment, we are very satisfied with the results obtained with this intervention [18, 34].

Therefore, this study showed that resistance exercise training, three times a week, 20 min a day, totaling 1 h weekly for 4 months (18 weeks), was no more effective for the intervention group than for the control group for managing fatigue. The same result was found for secondary outcomes measured in this study. However, for perceived fatigue control and all other outcomes, positive results of the exercise programs were observed for both the intervention and control groups. This study protocol was elaborated from previous studies demonstrating the positive effects of exercise based on resistance training in the workplace to decrease musculoskeletal complaints [13, 20]. Few studies have emphasized the effect of resistance exercise training on perceived fatigue control with high methodological quality.

This study did not show greater benefit of one modality over another and allows us to conclude that both can be beneficial, the implementation of which remains at the discretion of the company. The exercise program was performed for 4 months. (18 weeks). This period would physiologically be enough for there to be an increase of muscle strength, since the intervention group had a load increase of only 20% over the individuals’ initial loads, which were 30% of 1RM. Although positive results have been demonstrated within groups, our findings could also be explained by moderate job demand and exposure of workers included in the study [21, 22].

The intervention was conducted in the work environment and, for this reason, low loads were used during the protocols’ execution. The employees had no rest intervals after the 20 min exercise periods, instead going directly back to their work. Causing fatigue or pain in the volunteers during their work, which could compromise their performance and productivity, was to be avoided and, besides that, compensatory movements were avoided with the use of this protocol. In addition, the benefits promoted by the exercise protocols went beyond the control of fatigue, contributing positively to changes in general life habits and health status both in the workplace and in the outside world.

In the present study, the control group still participated in an exercise program (as opposed to serving as a control by not performing any exercise) using constant loads from elastic bands with moderate resistance, which generated a muscular response. All workers in the control group, similar to the intervention group, positively benefited from a minimal effect reducing their perceived fatigue control. Other studies have shown that physical exercise in the workplace performed at least three times a week with moderate to vigorous intensity have presented broad health benefits [35, 36], which our results corroborate. Further studies may elucidate opportunities to achieve even greater health benefits since, according to the recommendations of the American College of Sports Medicine [37], the combination of different intensities of exercise is ideal for the maintenance and improvement of the musculoskeletal, cardiovascular, and neuromotor systems.

Regarding the secondary outcome measures, there was a significant decrease in musculoskeletal complaints in almost all body segments with the greatest improvements in the neck, shoulder, spine, and knees as well as reductions in body fat percentage. In addition, an increase of physical activity level, general health condition (including blood pressure, heart rate, and body mass index, among others), and productivity was observed in the within-groups comparisons. At the four-month evaluation, the workers demonstrated improvements in general life habits. They reported decreased perceived fatigue, decreased musculoskeletal complaints, and being more willing and prepared for their jobs.

A recent systematic review demonstrated strong evidence for the effectiveness of resistance training in the upper extremities for the prevention of musculoskeletal symptoms [9]. In addition, a randomized controlled study involving women with musculoskeletal disorders in the neck and upper limbs who performed manual tasks with moderate risk showed that, after 6 months of participating in a twice per week workplace exercise program, they experienced significant reductions of pain in the neck and upper limbs as well as improvements in grip strength [8], thus showing the importance of exercise for all populations of workers.

Only the elbow and ankle regions did not present significant results. The hip/thigh region also did not present significant results between groups but did end with zero scores for musculoskeletal complaints after the exercise program. While this emphasizes that exercise performed in the work environment is able to reduce musculoskeletal symptoms, the beneficial effect depends on the characteristics of the exercise programs performed. For example, the exercise should occur for periods of more than 10 weeks, include exercises performed with some type of resistance, and be supervised [13].

Periodic physical fitness assessments including body composition, muscular strength, muscular endurance, balance tests, and blood pressure are important to identify health problems that could be ameliorated through workplace exercise programs [38]. The physical fitness outcomes help companies to implement new interventions for these workers, such as programs to reduce sedentary behaviors and increase physical activity during work [10].

The workers adhered strongly to the exercise program proposed in our study. We credit this result to the support of the managers and to the commitment of the team of professionals who conducted the exercise program and motivated the workers daily. Physical exercise programs in the workplace have been demonstrated to have more positive effects when the workers are motivated to perform them and have high adherence to the exercise program [13]. Similar to our study, Zebis et al. observed high adherence of participants (85%), with 63% of participants performing exercise two to three times a week, 15% performing exercise one to two times a week, and 7% performing exercise once a week [39]. Experienced instructors and small training groups may have been the reason for the success of the physical exercise program in the workplace [39]. Regarding this, research on adherence to physical exercise programs in the workplace conducted in Brazil in cooperation with the Industrial Social Service (SESI) observed that the participation of workers varies between 40 and 50% and depends on the presence of a team of instructors, manager involvement, and positive reinforcement [40].

To carry out this study, there were no high costs for the company. The company bought some of the office supplies to record the execution of the exercises and paid for the banner and notices that were fixed in the sectors. It also provided a computer room and space for worker assessments. The ten professionals who guided the physical exercise program volunteered for this research and came from the university where one of the researchers works.

Based on the study results, it is possible to recommend the implementation of a resistance exercise program in the workplace. Our findings showed that both programs were equally effective in perceived fatigue control, although the use of elastic bands for resistance is simpler, easier, therefore more feasible in daily.

### Limitations and strengths

The initial difficulties of this study were convincing managers about the benefits of physical exercise in the workplace and obtaining permission to interrupt the work three times a week for 20 min for physical exercise. Brazil’s economic situation during the study period led to a contraction of jobs and an increase in layoffs. Although the layoffs did not reach the workers participating in the project due to negotiations with the managers, the climate in the dairy industry generated a lot of stress among them and greater work demands. General complaints have increased among many of the workers, and many have begun to reduce the length of their lunch and snack breaks for fear of losing their jobs.

As a positive aspect, the managers’ confidence in the scientific data presented by the research team, regarding the effectiveness of the physical exercise program on workers` health. In addition to the proposal being considered innovative since it had not been implemented previously, it was a facilitator so that the project project could be implemented and completed.

Exercise in the workplace is beneficial for both the workers and companies due to the important changes in workers’ general health conditions. This study demonstrated that exercise programs in the workplace are possible and bring benefits but also require greater investments. Therefore, it is important for subsequent studies to analyze the cost-effectiveness of these programs.

## Conclusion

The implementation of resistance exercise training in the dairy industry demonstrated that physical exercise in the workplace during the work schedule with progressively increasing loads was not more effective than resistance exercise training with constant loads in the perceived fatigue control, muscle strengh and other evaluated outcomes after a 4 month follow-up. All groups showed significant improvements after the exercise program, demonstrating the effectiveness of this practice in the work environment for health promotion.

## Data Availability

The databases are available from the corresponding author on reasonable request.

## Abbreviations

RCT: Randomized Controlled Trial
QEC: Quick Exposure Check
IG: Intervention group
CG: Control group
1-RM: One-repetition maximum contraction
SESI: Industrial Social Service
Br-NFR: Brazil - Need for Recovery Scale
HPQ: Health and Work Performance Questionnaire
PRE: Progressive Resistance Exercise
CWE: Compensatory Workplace Exercise
BMI: Body Mass Index
NMQ: Nordic Musculoskeletal Questionnaire
PAO: Physical activity level
ESL: Sport and exercise in leisure time
ELL: Exercise in leisure and locomotion
